# Academic motivation scale - reliability and validity evidence among undergraduate nursing students*

**DOI:** 10.1590/1518-8345.3848.3420

**Published:** 2021-04-12

**Authors:** Geisa Colebrusco de Souza, Everson Meireles, Vera Lúcia Mira, Maria Madalena Januário Leite

**Affiliations:** 1 Universidade Federal de São Paulo, Escola Paulista de Enfermagem, São Paulo, SP, Brazil.; 2 Universidade Federal do Recôncavo da Bahia, Centro de Ciências da Vida, Santo Antônio de Jesus, BA, Brazil.; 3 Universidade de São Paulo, Escola de Enfermagem, São Paulo, SP, Brazil.

**Keywords:** Motivation, Education, Nursing, Baccalaureate, Validation Studies, Reproducibility of Results, Factor Analysis, Statistical, Psychometrics, Motivação, Bacharelado em Enfermagem, Estudos de Validação, Reprodutibilidade dos Testes, Análise Fatorial, Psicometria, Motivación, Bachillerato en Enfermería, Estudios de Validación, Reproducibilidad de los Resultados, Análisis Factorial, Psicometría

## Abstract

**Objective:**

to assess the evidence of validity and reliability of the academic motivation scale (AMS) based on the internal structure.

**Method:**

this is a methodological study with 205 undergraduate nursing students. Dimensionality/internal structure of the AMS was assessed using factor analysis in the context of exploratory structural equation modeling (ESEM) and reliability of the factors was assessed by Cronbach’s alpha (α) coefficient and composite reliability (CR) coefficient.

**Results:**

acceptable fit indexes were obtained (CFI = 0.92; RMSEA = 0.07; SRMR = 0.06) for a three-dimensional model: intrinsic motivation (10 items; α = 0.84; CR = 0.86); extrinsic motivation (8 items; α = 0.84; CR = 0.90); and demotivation (4 items; α = 0.84; CR = 0.88). A significant correlational pattern was found for the motivation continuum.

**Conclusion:**

the dimensionality analysis for the AMS presented a model with three factors: intrinsic motivation, extrinsic motivation and demotivation, and was considered a reduced alternative to the original version of seven factors. This study helped assess the validity of the measurement instrument and its theory refinement; further studies should be conducted to assess its invariance property.

## Abstract (pt)

**Objetivo:**

avaliar as evidências de validade e a confiabilidade da Escala de Motivação Acadêmica (EMA) com base na estrutura interna.

**Método:**

pesquisa metodológica realizada com 205 estudantes de graduação em Enfermagem. A dimensionalidade/estrutura interna da EMA foi avaliada por meio de análises fatoriais, no contexto de Modelagens de Equações Estruturais Exploratórias (ESEM) e a confiabilidade dos fatores foi aferida pelos coeficientes alfa de Cronbach (α) e Confiabilidade Composta (CC).

**Resultados:**

foram obtidos índices de ajuste aceitáveis (CFI = 0,92; RMSEA = 0,07; SRMR = 0,06) para um modelo tridimensional: Motivação Intrínseca (10 itens; α = 0,84; CC = 0,86); Motivação Extrínseca (08 itens; α = 0,84; CC = 0,90); e Desmotivação (04 itens; α = 0,84; CC = 0,88). Encontrou-se padrão correlacional significativo para o *continuum* de motivação.

**Conclusão:**

a análise da dimensionalidade da EMA alcançou um modelo de três fatores: Motivação Intrínseca, Motivação Extrínseca e Desmotivação, sendo considerada uma alternativa parcimoniosa à versão original de sete fatores. Este estudo contribuiu para a avaliação da validade do instrumento de medida e seu refinamento teórico, apontando a necessidade de que investigações futuras avaliem sua propriedade de invariância.

## Abstract (es)

**Objetivo:**

evaluar las evidencias de validez y confiabilidad de la Escala de Motivación Académica (EMA) en base a la estructura interna.

**Método:**

estudio metodológico realizado con 205 estudiantes del curso de Enfermería. La dimensionalidad/estructura interna de la EMA fue evaluada por análisis factorial contextualizado en los Modelos de Ecuaciones Estructurales Exploratorias (ESEM). La confiabilidad de los factores fue comprobada por los coeficientes Alfa de Cronbach (α) y Confiabilidad Compuesta (CC).

**Resultados:**

fueron obtenidos índices de ajuste aceptables (CFI = 0,92; RMSEA = 0,07; SRMR = 0,06) para un modelo tridimensional: motivación intrínseca (10 ítems; α = 0,84; CC = 0,88), motivación extrínseca (08 ítems; α = 0,84; CC = 0,90) y desmotivación (04 ítems; α = 0,84. CC = 0,88). Se encontró estándar correlacional significativo para el *continuum* de motivación.

**Conclusión:**

el análisis de dimensionalidad de EMA alcanzó un modelo de tres factores: motivación intrínseca, motivación extrínseca y desmotivación, considerándoselo una alternativa simplificada de la versión original de siete factores. El estudio contribuyó evaluando la validez del instrumento de medición en su refinamiento teórico, destacando la necesidad de que su invariancia sea evaluada en investigaciones futuras.

## Introduction

According to the self-determination theory (SDT), motivation can be analyzed as a complex and multidimensional theoretical construct. The types of motivation can explain the different reasons why people act in a certain way, based on different types of regulation and causal locus of behavior. Motivation is considered autonomous when an action takes place due to a genuine interest in the activity, while the most controlled types of motivation occur when there is internal or external pressure to engage in an activity. The SDT also includes demotivation, that is, the absence of any type of motivation^(^ 1 ^-^ 5 ^)^.

Based on the SDT, Canadian researchers developed the *Echelle de Motivation en Education* (EME) in French^(^ 6 ^)^, which was translated into English, validated and renamed as the Academic Motivation Scale (AMS)^(^ 7 ^)^. This scale was developed to measure the motivation level of students and their self-perception of the reasons for engaging in an activity^(^ 6 ^-^ 8 ^)^. In the academic context, students are expected to have the most autonomous types of motivation, as studies have demonstrated positive relations between these autonomous types and student performance^(^ 3 ^-^ 5 ^)^.

In comparison with the theoretical construct of the SDT, the scale included seven factors of specific taxonomy for the educational context: intrinsic motivation to know, to accomplish things, and to experience stimulation; extrinsic motivation by external, introjected, and identified regulation. Factor seven refers to demotivation. Extrinsic motivation by integrated regulation was suppressed in the AMS because, in the factor analysis, it is not distinguished from motivation by identified regulation^(^ 7 ^-^ 8 ^)^.

The AMS has been translated into several languages^(^ 9 ^-^ 13 ^)^ and used in different educational contexts^(^ 14 ^)^ and different times, either in its original version with all items or versions including new items, which resulted in a modified instrument^(^ 15 ^-^ 16 ^)^. Several studies have assessed its psychometric properties^(^ 6 ^-^ 7 ^,^ 9 ^-^ 10 ^,^ 12 ^-^ 21 ^)^, confirming the theoretical model with seven factors through a confirmatory factor analysis (CFA)^(^ 6 ^-^ 7 ^,^ 9 ^-^ 10 ^,^ 12 ^-^ 15 ^,^ 17 ^-^ 19 ^)^ or rejecting it and presenting alternative models^(^ 11 ^,^ 16 ^,^ 20 ^-^ 21 ^)^ with a different number of factors. Some of these studies did not demonstrate a correlation between the subscales (motivation continuum)^(^ 7 ^-^ 8 ^,^ 18 ^-^ 20 ^,^ 22 ^)^, i.e., no simple, positive strong correlation pattern was found between the adjacent types of motivation^(^ 1 ^-^ 2 ^)^.

In this sense, the first assessment of the AMS dimensionality showed inconsistencies in the exploratory factor analysis (EFA) and did not reproduce the theoretical model with seven subscales, with eigenvalues above one in at least one of the factors^(^ 6 ^)^. In the scale applied in Canada, the CFA admitted the 7-factor structure of the theoretical model inadequately reproduced the observed covariance matrix; however, fit indexes increased after the inclusion of 26 residual correlations based on the modification indexes^(^ 7 ^)^.

Later, the AMS showed concurrent validity with other motivation measurement instruments, with an appropriate correlation between the subscales regarding the hypothesis of motivation continuum postulated in the SDT, except for intrinsic motivation for stimulating activities, which showed a weak correlation^(^ 8 ^)^.

In Brazil, the scale was translated into Portuguese^(^ 23 ^)^ and applied to medical students; however, no robust analysis has assessed its dimensionality and reliability as an instrument to measure motivation. Although accepted in national studies^(^ 15 ^,^ 17 ^,^ 21 ^)^, since it was developed, new guidelines for the assessment of psychometric properties, translation and adaptation of instruments have been incorporated into the literature^(^ 24 ^-^ 25 ^)^. Therefore, processes must be adopted to evaluate its properties to ensure a valid and reliable scale for the measurement of academic motivation.

The stages of validation and evaluation of psychometric instruments must be performed in a continuous process to provide valid current reliable data about the measurement instruments^(^ 24 ^)^. In view of the above, this study aimed to assess the evidence of validity of the academic motivation scale based on the internal structure^(^ 26 ^)^ and the reliability indexes of the proposed dimensions of measurement.

## Method

This is a methodological study conducted with undergraduate nursing students from a public university in the state of São Paulo in the second half of 2014. It used a convenience sample, which consisted of students who were available to participate in the investigation.

Considering the scarcity of evaluations about the factor structure of the AMS in the Brazilian context, the author decided to use the version translated from English^(^ 7 ^)^ into Portuguese^(^ 23 ^)^, maintaining all items of the original version. The authors of this instrument authorized to use it for educational purposes^(^ 7 ^)^.

The original version of the AMS proposes to analyze motivation in the academic context, covering 28 propositions divided into seven subscales with four items each, scored on a Likert scale from 1 (no agreement) to 7 (total agreement), with a mean of 4 (moderate correspondence)^(^ 7 ^,^ 23 ^)^. A student indicates the agreement with a statement, allowing the calculation of each type of motivation based on the items linked with the proposed theoretical construct, according to Table 1.

**Table 1:** Type of motivation and items related to the original constructs of the academic motivation scale (AMS). São Paulo, SP, Brazil, 2014

Type of motivation (motivation continuum)		Items related to the original construct	Score
Intrinsic motivation	To know	02; 09; 16; 23	1 - 7
	To accomplish things	06; 13; 20; 27	1 - 7
	To experience stimulation	04; 11; 18; 25	1 - 7
Extrinsic motivation	Identified regulation	03; 10; 17; 24	1 - 7
	Introjected regulation	07; 14; 21; 28	1 - 7
	External regulation	01; 08; 15; 22	1 - 7
Demotivation	Absence of motivation	05; 12; 19; 26	1 - 7

Source: Developed by the author based on the 7-factor model originally proposed by Vallerand, et al.^(^6^,^^7^^)^ and translated into Portuguese by Sobral^(^23^)^

In addition to the score for each of the seven types of motivation, three different factors were defined at a higher order of the scale^(^ 6 ^-^ 8 ^,^ 17 ^,^ 21 ^)^: Intrinsic Motivation, Extrinsic Motivation, and Demotivation, based on the arithmetic mean calculation.

Data were collected at formal times of academic activity after obtaining the authorization of coordinators and professors of the course who indicated the best moment for the researcher to explain the study participation. The students who agreed to participate remained in the room to answer the instrument and sign an informed consent form (ICF), which was returned to the researcher. The instrument had no student identification to ensure data confidentiality.

Data were organized in Microsoft Office Excel spreadsheets and factor analyses were performed using the exploratory structural equation modeling (ESEM)^(^ 27 ^)^, whose source of information was a polychoric correlation matrix. The methods of weighted least squares means and variance (WLSMV) estimation and GEOMIN oblique rotation were adopted. These analyses were conducted using the Mplus 7^(^ 28 ^)^ and the number of factors to be extracted was indicated by parallel analyses^(^ 29 ^)^. The criterion for maintaining an item in the instrument was defined *a priori*: saturation of factor load ≥0.40 and item-total correlation ≥0.40.

Estimated factor solutions were evaluated based on theoretical reasonability, interpretation of factors according to theoretical assumptions^(^ 7 ^)^, and degree of factor model adjustment to the empirical data. The following criteria were considered: a comparative fit index (CFI) above 0.90 indicated good adjustment; root-mean-square error of approximation (RMSEA) and standardized root-mean-square residual (SRMR) values below 0.08 indicated adjustments in these two residual indices, and values below 0.06^(^ 30 ^-^ 31 ^)^ were considered desirable. Factor reliability was assessed by Cronbach’s alpha and composite reliability coefficients, with values of 0.70 or above considered satisfactory indicators in exploratory studies^(^ 32 ^-^ 33 ^)^.

This study was approved by the Research Ethics Committee (*Certificado de Apresentação de Apreciação Ética* - CAAE 45542415.7.0000.5392) and by those in charge representing the institution and was conducted according to the ethical practices for research with human beings.

## Results

This study analyzed 205 instruments, which were filled out by 68.5% of all students enrolled in the nursing course. Of these, 32.7% were from the 1st year of the course (N=67), 26.8% from the 2^nd^ year (N=55), 22.9% from the 3^rd^ year (N=47), and 17.6% from the 4^th^ year (N=36). Mean age of the students was 21.7 (SD=3.81), median of 21 (range 18-45 years), and 62.4% (N=128) were aged 18 to 22 years. Most were female students, corresponding to 88.29% (N=181).

**Figure 1:**
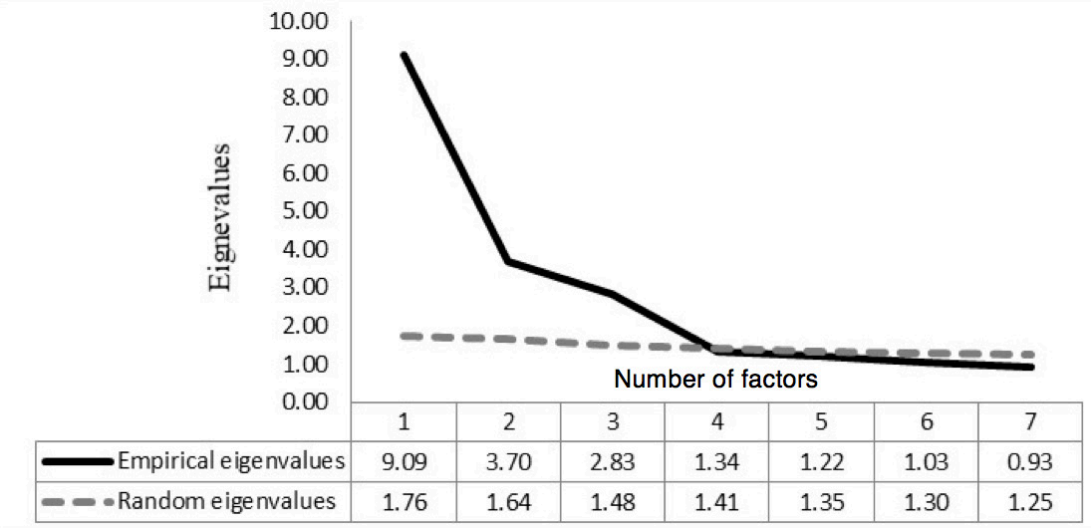
Sediment graph of the parallel analysis for the academic motivation scale. São Paulo, SP, Brazil, 2014

Initially, the results of parallel analyses indicated the pertinence of extracting up to three factors and did not support the extraction of seven factors, as proposed in the original theoretical model illustrated in Figure 1. After the third factor, any values in a random data matrix would be able to produce eigenvalues above the empirical eigenvalues.

Considering this indication, factor analyses were performed with extraction of one, two and three factors. The adjustment results of these models were: one-dimensional model (CFI = 0.68; RMSEA = 0.14; SRMR = 0.16); 2-factor model (CFI = 0.82; RMSEA = 0.11; SRMR = 0.11), and 3-factor model (CFI = 0.92; RMSEA = 0.07; SRMR = 0.06). Only the 3-factor solution demonstrated an acceptable fit according to the criteria indicated for the structural model (RMSEA <0.08; SRMR <0.06; and CFI >0.90). Also, the 3-factor solution came close to the theoretical expectations after grouping the items, according to the original model of the scale, which provides three factors of a higher order (Intrinsic Motivation, Extrinsic Motivation and Demotivation).

Therefore, regarding the internal structure, the AMS could be represented in this study by three factors. Table 2 shows the estimated psychometric parameters for the items/factors.

**Table 2:** Factor matrix of the academic motivation scale extracted by exploratory structural equation modeling (ESEM). São Paulo, SP, Brazil, 2014

Item	F1* Extrinsic motivation	F2* Intrinsic motivation	F3* Demotivation	r^it†^
22	0.94	-0.20	-0.04	0.71
8	0.88	-0.13	-0.03	0.73
15	0.82	-0.31	0.01	0.61
1	0.68	-0.11	0.02	0.54
7	0.63	0.07	0.39	0.52
24	0.62	0.00	-0.41	0.42
14	0.61	0.21	0.19	0.61
28	0,54	0.30	0.28	0.53
11	-0.16	0.73	-0.21	0.59
9	0.10	0.72	-0.20	0.69
16	0.01	0.66	-0.40	0.60
18	-0.14	0.64	0.07	0.49
25	-0.15	0.64	0.04	0.47
20	0.10	0.58	-0.05	0.54
4	-0.09	0.57	-0.21	0.45
2	0.02	0.55	-0.30	0.50
6	0.32	0.52	-0.01	0.58
13	0.37	0.51	0.03	0.52
26	0.10	-0.02	0.93	0.63
5	-0.10	-0.12	0.78	0.58
19	0.09	-0.21	0.78	0.54
12	-0.01	0.10	0.69	0,48
Number of items	08	10	04	
Eigenvalue	9.09	3.70	2.83	
Cronbach's alpha (α)	0.84	0.84	0.71	
Composite reliability (CR)	0.90	0.86	0.88	
Average variance extracted (AVE)	0.53	0.38	0.64	

* Correlation coefficients estimated at the level of p ≤0.05;

† item-total correlation coefficient.

When analyzing the items grouped by AMS factors based on the original proposal presented in Table 1, each factor corresponded to a different type of motivation: Factor 1 corresponded to Extrinsic Motivation and grouped items were: 22, 08, 15, 01, 03, 10, 07, 24, 14, and 28. Factor 2 corresponded to Intrinsic Motivation, whose grouped items were: 11, 09, 16, 18, 25, 20, 04, 02, 06 and 13; and Factor 3 corresponded to Demotivation with the following grouped items: 26, 05, 19, and 12.

Of all 28 items in the original scale, 22 items were retained in the factor analysis. Three items (17, 21, 27) did not present factor loads equal to or greater than 0.40 in any of the three factors. Other three items (03, 10, 23) were excluded due to theoretical and empirical difficulty to establish factor dominance in explaining the items - item 23, for example, presented 0.43 saturation in Factors 2 and 3. Thus, when compared to the theoretical expectation of grouping into higher order factors of the items, as illustrated in Table 1, three items that belonged to the extrinsic motivation subscale by identified regulation (17, 03 and 10), an item that belonged to intrinsic motivation to accomplish things (27) and another belonging to intrinsic motivation to know (21) were excluded.

In this study, the findings related to reliability of factors (Cronbach’s alpha and composite reliability) of the groups of factors/types of motivation were, respectively: 0.84 and 0.90 for intrinsic motivation; 0.84 and 0.86 for extrinsic motivation; 0.71 and 0.88 for demotivation.

The three factors/types of motivation of the AMS were structurally related to each other in a significant matter, presenting different patterns. The relationship between Intrinsic and Extrinsic Motivation was positive and moderate (they shared about 20% of variance) and, the relationship between Demotivation and Intrinsic and Extrinsic Motivation was negative and weak (they shared 1% to 2% of variance, respectively), as indicated in Table 3. From the line of Extrinsic Motivation factor, the indicators of discriminant validity (√*VME*) are observed diagonally for the modeled latent factors.

**Table 3:** GEOMIN correlation and evidence of discriminant validity between modeled factors (N=205). São Paulo, SP, Brazil, 2014

Factors	F1*. Extrinsic Motivation	F2*. Intrinsic Motivation	F3*. Demotivation
F1. Extrinsic Motivation	0.73		
F2. Intrinsic Motivation	0.45	0.62	
F3. Demotivation	-0.15	-0.11	0.80

* Correlation coefficients estimated at the level of p ≤0.05

Although the AMS has been widely applied in its English version since its original proposal and considered one of the main instruments for measuring academic motivation in different countries, the Brazilian context lacked information about its dimensionality.

## Discussion

Specialized literature shows that psychometric instruments must undergo successive assessments of dimensionality^(^ 34 ^)^, i.e., their internal structure, to ensure reliable data collection. They are also recommended to different contexts and moments. The evaluation of the validity evidence, based on the internal structure, proved to be necessary to check whether measurement attributes corresponded to the theoretical attributes. Therefore, the first question was what the test measured in order to later use it and accept it as a valid instrument to measure the academic motivation of undergraduate nursing students.

The result of parallel analyses did not support the extraction into seven factors according to the theoretical proposal of the authors of the scale^(^ 6 ^-^ 7 ^)^, since the matrix of empirical data of this study could be reduced to a maximum of three factors based on criteria found in the literature^(^ 29 ^,^ 35 ^)^.

The factor analyses in this study partially reproduced the theoretical model. Most of the item/factor relationships foreseen in the higher order structure^(^ 6 ^-^ 8 ^)^ were observed and the factor loads estimated at the level of p ≤0.05 were considered “good” (above 0.55) and “excellent” (above 0.71), according to the proposed taxonomy^(^ 36 ^)^.

The EFA based on the ESEM^(^ 27 ^)^ aimed to find a factor matrix of the scale based on a spontaneous relationship between observable variables (items) and latent variables (factors) and presented robust evidence of the existence of a three-dimensional model with three types of motivation: one factor for intrinsic motivation, one for extrinsic motivation, and one for demotivation. The 3-factor model was used to prevent both overestimated factors and a larger number than adequate, which would produce non-parsimonious results, and underestimated factors and a lower number, thus causing loss of precious information^(^ 35 ^)^.

In the assessment of how all 28 items would interact, the configuration of three types/factors proved to be relevant to measure the motivation construct in the studied sample, in agreement with the SDT^(^ 1 ^,^ 2 ^)^. Thus, this study could attest a parsimonious alternative to the original version of the scale, with a valid reliable configuration in three factors of a higher order: Extrinsic Motivation, Intrinsic Motivation, and Demotivation.

The structure of seven and six factors was replicated in Brazil; however, the study used a modified version of the AMS for factor extraction and a principal component analysis (PCA)^(^ 18 ^)^, which may have inflated the loading of items^(^ 37 ^)^.

Since the first version of the AMS, new statistical tests have been developed to identify the validity of measurement instruments in terms of theoretical constructs. In addition to replicating the scale in different contexts and moments, proper statistical tests based on EFA and CFA must be adopted, with psychometric modeling techniques and updated software. In general, studies aiming to evaluate the psychometric properties of instruments indiscriminately use programs and tools of reliability coefficients and PCA. Although they are common in usual statistical programs, these tools are not necessarily the most adequate techniques^(^ 34 ^)^.

Another study conducted in Brazil evaluated the factor structure of the AMS through EFA^(^ 17 ^)^, with extraction of five factors and explanation of 61.8% variance, reproducing the structure model of the AMS in the subscales of extrinsic motivation and demotivation; however, the three subscales of intrinsic motivation were grouped into a single factor, similar to what occurred in this study. The theoretical model of seven factors was achieved only with CFA and absolute fit index RMSEA = 0.07; SRMR = 0.06; and incremental fit indexes TLI (Tucker-Lewis Index) = 0.92; CFI = 0.93 and was more adequate than the 5-factor model (RMSEA = 0.09; SRMR = 0.07; TLI = 0.90; CFI = 0.89). However, that study^(^ 17 ^)^ used Pearson’s matrix, which usually identifies relationships between metric variables. For the AMS, since it is an ordinal scale, its relations are better identified using the polychoric correlation coefficient. Pearson’s correlation tends to underestimate the correlation between items with ordinal/categorical responses and overestimate the number of factors in exploratory factor analyses^(^ 27 ^-^ 28 ^)^.

In short, the adoption of robust criteria for the extraction of factors could explain the difference in the results found in the number of factors extracted, both in the Brazilian context^(^ 15 ^,^ 17 ^,^ 21 ^)^ and scale application in English^(^ 7 ^,^ 19 ^,^ 38 ^)^ and other languages into which it has been translated, including Norwegian^(^ 9 ^)^, Spanish^(^ 10 ^)^, Chinese^(^ 12 ^)^, and Turkish^(^ 13 ^)^. It is noteworthy that most studies cited here used CFA in their assessments^(^ 7 ^-^ 10 ^,^ 12 ^-^ 15 ^,^ 17 ^-^ 19 ^)^.

As seen in the results of this study, other studies also proposed a new reconfiguration of the AMS by EFA, with the extraction of four factors: demotivation, extrinsic motivation by external regulation, extrinsic motivation by identified regulation, and intrinsic motivation, confirming the results by CFA, with proper validity and reliability and significant losses in models of one, two and three factors^(^ 39 ^)^. Other studies also pointed out inconsistencies in the number of factors, with extraction of five factors, in which the three types of intrinsic motivation were grouped into a single factor^(^ 20 ^,^ 40 ^)^, and extraction of three factors^(^ 41 ^)^. In a study with Lebanese medical students, PCA was used and the results showed the items converged into a 3-factor solution with explanation of 81.51% of total variance^(^ 11 ^)^.

In an assessment of the psychometric properties of the instrument with Chilean students of Dentistry^(^ 10 ^)^ and students of the health vocational course and social care course in Norway^(^ 9 ^)^ by CFA, the researchers confirmed a 7-factor model. In Argentina, two versions of the instrument were applied with high school and university students, and the results also reproduced the 7-factor model^(^ 14 ^)^. In a revised version in Spanish^(^ 16 ^)^ that included the subscale extrinsic motivation by integrated regulation in the AMS from the original version^(^ 6 ^)^, applied to Pedagogy students by CFA, the results showed acceptable adjustment indexes of the new structure, with eight factors^(^ 16 ^)^. The authors proposed the inclusion of a factor previously suppressed in the AMS^(^ 7 ^,^ 8 ^)^: extrinsic motivation by integrated regulation, to obtain an instrument that would allow the measurement of all motivation regulations proposed by SDT^(^ 1 ^-^ 2 ^)^ in the educational context in Spain.

When assessing the reliability of the scale factors used in this study, previous studies used the Cronbach’s alpha index, considering the following satisfactory values: 0.48 to 0.98^(^ 11 ^)^, 0.62 to 0.82^(^ 7 ^)^, 0.65 to 0.83^(^ 10 ^)^, 0.68 to 0.83^(^ 21 ^)^, 0.70 to 0.86^(^ 16 ^,^ 18 ^)^, 0.74 to 0.92^(^ 17 ^)^, 0.77 to 0.90^(^ 19 ^)^, 0.79 to 0.86^(^ 38 ^)^. The reliability of the factors by Cronbach’s alpha and the composite reliability were considered satisfactory in our study, according to the parameters reported in the literature^(^ 32 ^-^ 33 ^)^.

Regarding the pattern of item grouping, foreseen in the theoretical model in three factors of a higher order, it was empirically reproduced satisfactorily. Regarding the structural relationship between the factors/types of motivation, they were significantly related to different patterns. The relationship between intrinsic motivation and extrinsic motivation was positive and moderate (20% variance), indicating that, as one type of motivation increases, the other tends to increase as well. On the other hand, the relationship between demotivation and intrinsic and extrinsic motivation was negative and weak (1 to 2% variance, respectively), indicating that, as demotivation decreases, the factors/types of intrinsic and extrinsic motivation tend to increase^(^ 42 ^)^.

Regarding the existence of the self-determination continuum and motivation^(^ 1 ^,^ 6 ^-^ 8 ^)^ seen as a significant positive correlation between intrinsic and extrinsic motivation and a negative correlation between intrinsic motivation and demotivation, the results confirmed the findings of other studies^(^ 15 ^,^ 17 ^,^ 22 ^)^ and the theoretical model^(^ 1 ^,^ 2 ^)^. However, in the English version of the scale, most studies did not confirm this hypothesis^(^ 6 ^-^ 8 ^,^ 18 ^-^ 19 ^,^ 39 ^)^, suggesting this construct pattern did not exist or was limited^(^ 19 ^,^ 39 ^)^.

This study also assessed to what extent each modeled latent variable (that is, the AMS factors) was different and how it could be distinguished from the others. One way to obtain this evidence is to assess whether the square root of VME (√VME) is greater than the estimated variance shared between the constructs, i.e., greater than the correlation between latent factors. The results in Table 3 show evidence that all three factors were properly discriminated in this study, according to the literature^(^ 43 ^-^ 44 ^)^.

Regarding the content of the items, although it was not the objective of this study, the author of this study agreed with other authors who suggested that such content should be reviewed according to criteria recommended in the literature in order to make it more precise^(^ 17 ^)^ and that the scale has to be revised and updated as a measurement tool^(^ 15 ^,^ 18 ^-^ 19 ^,^ 22 ^)^. Although it is still one of the main instruments of academic motivation measurement today^(^ 45 ^,^ 46 ^)^, this study found the scale has to be improved, since the measurement attributes did not fully correspond to the theoretical attributes.

Specifically in the Portuguese version for the Brazilian context, the AMS should be explored more rigorously in the process of translation, cross-cultural adaptation, equivalence and frequency of terms, including reverse translation, not previously described^(^ 23 ^)^. A simple translation of the items does not ensure the maintenance of the original construct. Future studies could include a sequence with a cross-cultural adaptation and a more robust analysis of the factor structure^(^ 25 ^)^.

In the other countries where translated versions were used or in the contexts where the English version of the AMS was applied, the adoption of other analysis resources is suggested, that is, besides the CFA, ESEM is recommended as it allows the integration of structural equation modeling and EFA in a single analysis^(^ 27 ^)^.

Through ESEM this study presented different outcomes from previous studies that assessed the psychometric properties of the AMS with the determination of a 3-factor model according to the reference in the subject^(^ 27 ^)^. Although the three factors observed the theoretical groupings of higher order of the AMS (extrinsic motivation, intrinsic motivation, and demotivation), it was not possible to show the scale refining into subfactors; in other words, there was no differentiation of the extrinsic motivation subscales in three types (extrinsic motivation by identified regulation, introjected regulation, and external regulation) and no differentiation of intrinsic motivation in three types (intrinsic motivation to know, to accomplish things, and to experience stimulation). In practical and applied terms, for the studied sample, the construct of academic motivation was only identified in the superior theoretical grouping.

Study limitations referred to the application of the AMS in a single moment of the course from a single field (nursing), which limits the generalization to other courses and students. The validity of measurement instruments and, in this case, the AMS, must be tested with other samples, preferably using analysis methods and software that are more appropriate to the categorical data, as seen in the study scale, either in the Brazilian context or in other countries, with students from different courses and other educational levels, to expand the evidence of validity of the motivation measurement instrument.

## Conclusion

The dimensionality of the AMS produced a model with three factors/types of motivation, factors of a higher order in agreement with that proposed in the Self-Determination Theory and was considered a parsimonious alternative to the original version, which proposes the measurement of seven factors. The results showed that the AMS presents evidence of satisfactory reliability and validity for three types of motivation: intrinsic motivation, extrinsic motivation, and demotivation, with robust evidence of adjustment and proper discrimination.

The findings supported the existence of the autonomy continuum, with significant positive correlations between intrinsic motivation and extrinsic motivation and a negative correlation between intrinsic motivation and demotivation.

Although new evidence of the psychometric quality of the AMS has been obtained, with the limitation of measuring three different types of motivation among nursing students, investigations with larger and more diversified samples are required so that the invariance properties of the scale can be properly investigated.
